# Racial/Ethnic Differences in Early Detection and Screening for Chronic Kidney Disease Among Adults in Hawaii: A 10-Year Population Health Study

**DOI:** 10.5888/pcd17.200011

**Published:** 2020-08-20

**Authors:** Merle R. Kataoka-Yahiro, James Davis, Connie M. Rhee, Linda Wong, Glen Hayashida

**Affiliations:** 1 School of Nursing and Dental Hygiene, Department of Nursing, University of Hawaii at Manoa, Honolulu, Hawaii; 2 Office of Biostatistics and Quantitative Health Sciences, John A. Burns School of Medicine, University of Hawaii at Manoa, Honolulu, Hawaii; 3 Division of Nephrology, Hypertension, and Kidney Transplantation, University of California, Irvine, Orange, California; 4 John A. Burns School of Medicine, University of Hawaii at Manoa, Honolulu, Hawaii; 5 National Kidney Foundation of Hawaii, Honolulu, Hawaii

## Abstract

**Introduction:**

Native Hawaiian and Asian American populations are the most understudied racial/ethnic groups in chronic kidney disease (CKD) research. The objective of our study was to describe sociodemographic and comorbidity risk factors of chronic kidney disease among 2,944 community-dwelling Native Hawaiian, Filipino, Chinese, Japanese, and non-Hispanic white participants who attended the National Kidney Foundation of Hawaii Kidney Early Detection Screening program during 2006–2017.

**Methods:**

We used multivariable logistic regression models to examine the association between age, sex, race/ethnicity, and the major risk factors for CKD (diabetes, hypertension, cardiovascular disease, hypercholesterolemia, overweight and obesity, and smoking) with elevated urine albumin to creatinine ratio (ACR) among adults aged 18 or older in 5 racial/ethnic groups in Hawaii: Native Hawaiian, Filipino, Chinese, Japanese, and non-Hispanic white.

**Results:**

In the age- and sex-adjusted model, Native Hawaiian participants were significantly more likely than non-Hispanic white participants to have an ACR of 30.0 mg/g or more (odds ratio [OR] = 1.50; 95% CI, 1.15–1.95; *P* = .003). In the model that adjusted for CKD risk factors, the difference between Native Hawaiian and non-Hispanic white participants became nonsignificant (OR = 1.27; 95% CI, 0.96–1.69; *P* = .09]). The higher prevalence of chronic conditions among Native Hawaiians partially explained their higher risk of having an elevated ACR. Filipinos had significantly higher odds than non-Hispanic whites of elevated ACR in the age- and sex-adjusted model (OR = 1.44; 95% CI, 1.14–1.84; *P* = .003) and after adjustment for CKD risk factors (OR = 1.36; 95% CI, 1.06–1.74; *P* = .01).

**Conclusion:**

Culturally targeted interventions are needed to improve health outcomes among Native Hawaiians and Asian Americans, particularly Filipinos, with CKD. Such interventions should focus on early kidney disease management so that disease progression can be delayed.

**Summary**

**What is already known on this topic?**

Native Hawaiian and Asian American populations are the most understudied racial/ethnic groups in research on chronic kidney disease (CKD).

**What is added by this report?**

This population health study provides substantive cross-sectional findings on Native Hawaiians and Asian American subgroups based on sociodemographic and comorbidity risk factors of early stages of CKD. Racial/ethnic disparities in CKD outcomes exist for Native Hawaiians and Filipino Americans.

**What are the implications for public health practice?**

Identification of CKD risk factors and CKD outcomes among Native Hawaiians and Asian Americans, particularly Filipinos, could improve health outcomes among these populations.

## Introduction

Asian Americans are the fastest growing racial/ethnic group in the United States; 17.6 million Asian Americans and 1.3 million Native Hawaiians resided in the United States as of 2018 (1). The prevalence of end-stage renal disease (ESRD) is 9.5 times greater among the Native Hawaiian population and 1.3 times greater among the Asian American population than among the non-Hispanic white population (2). Medicare spending for ESRD in the United States was $35 billion in 2016, and the costs of ESRD will continue to escalate according to the projected population growth of Native Hawaiians and Asian Americans in the United States during the next 50 years.

Interventions aimed at reducing chronic kidney disease (CKD) have not always translated into improved outcomes among Native Hawaiian and Asian American populations (3,4). In the United States, CKD is more common in racial/ethnic minority populations than in the non-Hispanic white population, but of these racial/ethnic minority groups, Native Hawaiians and Asian Americans are the least studied. Previous studies of CKD-related disparities among Native Hawaiians and Asian Americans provided limited information because of the way data on these populations were categorized (4–6). These studies, for example, aggregated all data on Asian Americans, combined data on Native Hawaiians with data on Asian Americans, merged data on Native Hawaiians and Asian Americans into an “other” category, or excluded data on these populations. Although our understanding of the risk factors for and sequelae of CKD among other racial/ethnic minority populations in the United States, such as the African American and Hispanic populations, have progressed, only a few studies among Native Hawaiians (7–11) and Asian Americans (4–11) have explored the association of CKD risk factors, particularly for the early stages of CKD.

Hawaii is a racially/ethnically diverse state; more than half of the population is of Native Hawaiian or Asian descent, and it is thus an ideal setting for studying these populations. The objective of our study was to describe the sociodemographic and comorbidity risk factors of CKD and CKD outcomes of community-dwelling Native Hawaiians and Asian Americans who attended the National Kidney Foundation of Hawaii (NKFH) Kidney Early Detection Screening (KEDS) program.

## Methods

This cross-sectional population health study of sociodemographic and comorbidity CKD risk factors (diabetes, hypertension, cardiovascular disease, hypercholesterolemia, overweight and obesity, and smoking) used health screening data from the NKFH KEDS program from January 1, 2006, to December 31, 2017. We included KEDS program participants in our analyses if they were 1) aged 18 years or older and 2) self-reported as Native Hawaiian, Filipino, Chinese, Japanese, or non-Hispanic white race/ethnicity. We excluded participants who reported a mixed race/ethnicity because of small numbers. Our analytic sample consisted of 2,944 adults. The study was approved by the University of Hawaii Office of Research Compliance.

### Setting

NKFH KEDS is a statewide grassroots community outreach health screening initiative adapted from a national program, Kidney Early Evaluation Program (KEEP). KEDS program intake form and data collection protocols were established in 2006 and were followed during the study period (12). A KEDS health screening event consisted of participants rotating through 5 stations, in the following order: Station 1, registration; Station 2, physical measurements; Station 3, urinalysis; Station 4, blood collection; and Station 5, exit interview (clinician consultation). Participants spent approximately 25 to 60 minutes to complete all 5 stations. 

At Station 1, registration, participants signed in and completed the intake assessment form. Community volunteers assisted participants with visual impairments or language barriers. At Station 2, physical measurements, student or professional volunteers measured blood pressure and height and weight. A Tanita BWB-800 digital weighing scale was used to measure height and weight. At Station 3, urinalysis, volunteers provided participants with a specimen cup and instructions on how to provide a clean-catch urine sample. Urine specimens were processed by using a Bayer Clinitek 50 urine chemistry analyzer or a Siemens Clinitek Status + Analyzer; a Bayer/Siemens Diagnostics Microalbumin Reagent test strip was used for testing albumin, creatinine, and albumin to creatinine ratio (ACR). At Station 4, blood collection, professionals skilled in phlebotomy collected venous or capillary blood specimens. Venous blood draw specimens were transported via couriers to a local laboratory for processing. At Station 5, the exit interview, clinicians conducted brief (approximately 5–10 minutes) interviews with participants and reviewed screening results. General recommendations and education on risk factors for CKD were also provided. Participants with concerns or abnormal results were advised to follow up with their primary care providers. Venous blood specimen results were mailed to the participants’ homes 7 to 10 days after the screening.

### Data collection

We collected information on participants’ sociodemographic characteristics, including age, sex (female, male), race/ethnicity (Native Hawaiian, Filipino, Chinese, Japanese, and non-Hispanic white), zip code, and health risk factors for the purposes of program planning and evaluation. We collected information on the following clinical risk factors for CKD: a medical history of diabetes (type 1 and 2), hypertension, cardiovascular disease, or hypercholesterolemia; or a history of cigarette smoking (current or past). For the purposes of this study, we used the self-reported data on the history of risk factors; we did not use laboratory results. We used laboratory results only for ACR. We calculated body mass index (BMI) as weight in kilograms divided by height in meters squared and used the following BMI categories (13): normal or underweight, <25.0; overweight, 25.0–29.9; obese, ≥30.0. We classified ACR as normal (<30 mg/g) or abnormal (≥30 mg/g) (14).

### Data analysis

We generated descriptive statistics for the sample studied, including frequencies and percentages for categorical variables and means and standard deviations (SDs) for continuous variables. We also compared means and SDs (or percentages) of characteristics by race/ethnicity. We assessed significant differences among race/ethnicities by using analysis-of-variance for continuous variables and χ^2^ tests for categorical variables. The KEDS program enrolled 4,029 participants from 2006 through 2017. Of these, 3,147 were eligible for our study based on their race/ethnicity. We excluded 203 (6.5%) participants because of missing data on one or more variables. The analysis population consisted of the remaining 2,944 KEDS participants. We performed all analyses in SAS version 9.4 (SAS Institute Inc). A *P* value of <.05 was considered significant.

We used logistic regression analyses to examine the association between the predictors of race/ethnicity, age, sex, diabetes, hypertension, cardiovascular disease, hypercholesterolemia, and overweight or obesity, smoking, and an ACR of 30 mg/g or more. We treated age as a continuous variable and the other covariates as dichotomous variables. We used logistic regression analyses with the following levels of covariate adjustment: Model 1, an unadjusted model that included race/ethnicity; Model 2, adjusted for race/ethnicity, age, and sex; and Model 3, adjusted for covariates in Model 2, as well as diabetes, hypertension, cardiovascular disease, hypercholesterolemia, overweight or obesity, and smoking.

## Results

The mean age of the study sample was 57.5 (SD, 16.8) years; 61.9% were women (Table 1). Of the 2,944 participants, 37.6% were non-Hispanic white; 11.9%, Native Hawaiian; 19.3%, Filipino; 7.1%, Chinese; and 24.1%, Japanese. In the study sample, 38.6% reported a history of hypertension; 31.3%, a history of hypercholesterolemia; 17.5%, a history of diabetes; and 7.1%, a history of cardiovascular disease. Of the study sample, 16.1% reported current or past cigarette smoking, 76.4% had normal weight or were underweight, 14.7% were overweight, and 8.9% were obese. We found an ACR of 30 mg/g or more in 16.8% of the study sample.

**Table 1:** Characteristics of Study Sample (N = 2,944), by Race/Ethnicity, National Kidney Foundation of Hawaii Kidney Early Detection Screening Program, 2006−2017

Characteristic	All, No. (%)	By Race/Ethnicity					
		Non-Hispanic White	Native Hawaiian	Filipino	Chinese	Japanese	*P* Value^a^
No. (%)	2,944 (100.0)	1,107 (37.6)	350 (11.9)	569 (19.3)	209 (7.1)	709 (24.1)	—
Age, mean (SD), y	57.5 (16.8)	55.1 (16.4)	55.4 (16.1)	56.5 (16.2)	57.5 (17.8)	63.1 (16.6)	<.001
Female	1,821 (61.9)	56.6%	64.3%	65.4%	62.7%	65.7%	<.001
Current or past cigarette smoking	474 (16.1)	18.0%	24.3%	12.1%	9.6%	14.2%	<.001
Medical history of hypertension	1,135 (38.6)	30.1%	48.3%	45.5%	35.4%	42.3%	<.001
Medical history of hypercholesterolemia	922 (31.3)	25.2%	33.7%	35.0%	33.5%	36.1%	<.001
Medical history of diabetes	515 (17.5)	11.9%	26.9%	22.3%	12.9%	19.0%	<.001
Medical history of cardiovascular disease	210 (7.1)	7.2%	8.9%	5.4%	5.3%	8.0%	.20
Albumin to creatinine ratio ≥30 mg/g	495 (16.8)	13.5%	22.0%	20.0%	12.9%	17.9%	<.001
**Body mass index (BMI), kg/m^2^**							
Normal and underweight (<25.0)	2,248 (76.4)	72.3%	49.7%	81.9%	90.9%	87.2%	<.001
Overweight (BMI, 25.0–29.9)	434 (14.7)	16.7%	24.6%	14.2%	7.2%	9.4%	
Obese (BMI, ≥30.0)	262 (8.9)	11.0%	25.7%	3.9%	1.9%	3.4%	

a Significant differences by race/ethnicity assessed by using analysis-of-variance for continuous measures and χ^2^ tests for categorical variables.

All independent variables, except cardiovascular disease, differed significantly by race/ethnicity (Table 1). Japanese participants were the oldest (63.1 [SD, 16.6] y), and non-Hispanic white participants had the lowest proportion of women (56.6%). Native Hawaiians had high health-risk profiles; they were the most likely to smoke (current or past); have hypertension, diabetes, or cardiovascular disease; and to be overweight or obese.

In unadjusted logistic regression models, Native Hawaiian participants were significantly more likely than non-Hispanic white participants to have an ACR of 30.0 mg/g or more (odds ratio [OR], 1.30; 95% CI, 1.01–1.69; *P* = .045). This unadjusted difference may be partially explained by differences in age and sex in the study population. With age and sex adjustment, effect estimates for Native Hawaiian and Filipino participants became stronger; Native Hawaiian participants (OR, 1.50; 95% CI, 1.15–1.95; *P* = .003) and Filipino participants (OR, 1.44; 95% CI, 1.14–1.84; *P* = .003) were significantly more likely than non-Hispanic white participants to have an ACR of 30.0 mg/g or more (Table 2).

**Table 2:** Unadjusted and Age-Adjusted, Sex-Adjusted, and Multiple-Adjusted Models of Having an Albumin to Creatinine Ratio of ≥30.0 mg/g by Sociodemographic and Comorbidity Risk Factors of Chronic Kidney Disease in a Study Sample (N = 2,944), National Kidney Foundation of Hawaii Kidney Early Detection Screening Program, 2006−2017^a^

Variable	Unadjusted Model	Age- and Sex-Adjusted Model	Multiple-Adjusted Model
**Age**	—	1.03 (1.02–1.03) [<.001]	1.03 (1.02–1.03) [<.001]
**Female**	—	0.75 (0.62–0.92) [.006]	0.79 (0.64–0.97) [.02]
**Race/ethnicity**			
Non-Hispanic white	1 [Reference]	1 [Reference]	1 [Reference]
Native Hawaiian	1.30 (1.01–1.69) [.045]	1.50 (1.15–1.95) [.003]	1.27 (0.96–1.69) [.09]
Filipino	1.25 (0.99–1.58) [.06]	1.44 (1.14–1.84) [.003]	1.36 (1.06–1.74) [.01]
Chinese	0.77 (0.57–1.05) [.10]	0.85 (0.62–1.17) [.32]	0.88 (0.64–1.20) [.42]
Japanese	1.25 (0.99–1.57) [.06]	1.19 (0.94–1.51) [.16]	1.18 (0.92–1.50) [.19]
**Health characteristics**			
Current or past cigarette smoking (vs no history)	—	—	0.99 (0.75–1.30) [.94]
Medical history of hypertension (vs no history)	—	—	1.44 (1.16–1.79) [.001]
Medical history of hypercholesterolemia (vs no history)	—	—	0.83 (0.67–1.04) [.11]
Medical history of diabetes (vs no history)	—	—	1.42 (1.11–1.81) [.006]
Medical history of cardiovascular disease (vs no history)	—	—	1.20 (0.85–1.70) [.30]
**Body mass index (BMI), kg/m^2^**			
Normal and underweight (BMI, <25.0)	—	—	1 [Reference]
Overweight (BMI, 25.0–29.9)	—	—	1.34 (1.02–1.76) [.04]
Obese (BMI, ≥30.0)	—	—	1.27 (0.88–1.82) [.20]

Abbreviation: —, does not apply.

a All values are odds ratio (95% CI) [*P* value].

In the third model, which adjusted for the major CKD risk factors, the ORs for Filipinos and Native Hawaiians were attenuated. Filipino participants remained significantly more likely than non-Hispanic white participants (OR, 1.36; 95% CI, 1.06–1.74; *P* = .01) to have an ACR of 30.0 mg/g or more, whereas the difference between Native Hawaiian and non-Hispanic white participants became nonsignificant (OR, 1.27; 95% CI, 0.96–1.69; *P* = .09). For Native Hawaiians, adjustment for major CKD risk factors reduced the relative odds of having an ACR of 30.0 mg/g or more by 15.3% (1.50 – 1.27/1.50).

After accounting for differences in age and sex, the differences between Japanese participants and non-Hispanic white participants became smaller (from an OR of 1.25 in Model 1 to an OR of 1.19 in Model 2), whereas the differences between Native Hawaiian and Filipino participants and non-Hispanic white participants became larger (from an OR of 1.30 to an OR of 1.50 among Native Hawaiians and from an OR of 1.25 to an OR of 1.44 among Filipinos). In comparisons that accounted for major chronic diseases, the ORs comparing Filipino and Native Hawaiian with non-Hispanic white participants decreased.

## Discussion

In this 10-year population health study of community-dwelling Native Hawaiians and Asian American subgroups who attended the NKFH KEDS program, we examined the association of racial/ethnic groups, sociodemographic characteristics, and comorbid CKD risk factors with markers of early kidney damage, as measured by abnormal ACR levels. Our findings suggest that Filipinos and Native Hawaiians are at higher risk of early kidney damage, manifested by abnormal levels of albuminuria and which may be partially explained by the presence of chronic diseases.

Independent variables in our study associated with abnormal ACR were older age, female sex, a history of diabetes or hypertension, and overweight status. In seminal work with Native Hawaiians and Asian Americans using 2001–2003 disaggregated subgroup data from the NKFH KEEP, Mau et al examined the association between key CKD risk factors and CKD outcomes (7). Previous cross-sectional studies of the NKFH KEDS program used data on community-dwelling participants in Wave 1 (2006–2012) to examine the relationship of CKD risk factors and CKD outcomes among Native Hawaiian, Filipino, Chinese, Japanese, and non-Hispanic white participants in Hawaii (4,9–11). ACR and/or urine albumin levels were used as predictors for CKD outcomes. Their findings indicated that nearly all Native Hawaiian and Asian American groups had a higher risk for CKD than the non-Hispanic white group during the early stages of CKD. Age (4,10,11), diabetes (4,9,10), hypertension (4,10), and obesity (9,10) were predictors also found to be independently associated with higher risk for CKD. The findings of previous cross-sectional studies using NKFH KEDS and KEEP were consistent with the findings of our study.

Native Hawaiians and Filipinos had the highest risk profiles for CKD in our study, which is consistent with studies in the current literature. Mau et al found Native Hawaiians participating in NKFH KEEP had the highest prevalence of CKD among the groups studied; their risk was 4 times the risk among the general US population (7). Wong et al found NKFH KEDS participants who were Pacific Islanders (including Native Hawaiians) with obesity, hypertension, and diabetes had a higher risk than Asian Americans (Filipinos, Chinese, Japanese) and non-Hispanic whites of kidney damage defined by elevated ACR and urine albumin levels (10). Cardiovascular disease and associated cardiovascular risk factors are the leading cause of death among Native Hawaiians, with a 4-fold higher rate among Native Hawaiians with diabetes (15,16). Uchima et al found that Native Hawaiians and Pacific Islanders had higher rates of diabetes compared with other races/ethnicities in Hawaii (17). In a study by Mau et al on the association of modifiable risk factors and left ventricular ejection fraction among hospitalized Native Hawaiians and Pacific Islanders, 25% were smokers, 45% used alcohol, 23% used methamphetamine, 72% had hypertension, 49% had diabetes, and 37% had a previous myocardial infarction (18). Nearly 60% had moderate to severe left ventricular ejection fraction, and their BMIs were high. Native Hawaiians and Pacific Islanders have the highest mean BMI for men and women in comparison to other racial/ethnic groups in Hawaii; this high BMI contributes to a higher risk for CKD. The high prevalence of chronic diseases among Native Hawaiians may contribute to the high prevalence of abnormal ACR in that population.

Our study found that Filipinos also were at higher risk than Native Hawaiians, Chinese, Japanese, or non-Hispanic whites for CKD, and the findings of our study are consistent with the literature. Choi et al examined racial/ethnic differences in the development of albuminuria, examining data on Filipinos separately from data on Asian Americans (6). Their study found that for parallel groups, Filipinos and Asians with diabetes developed albuminuria at higher rates than non-Hispanic whites and Hispanics with diabetes. The percentage of Filipino, Hispanic, and African American participants with hemoglobin A_1c_ levels of >8% was greater than the percentage of non-Hispanic white and Asian American participants; 11% of African American, 10% of Filipino, 9% of Asian American, 9% of Hispanic, and 8% of non-Hispanic white participants developed albuminuria. In fully adjusted models, Choi et al found that, in comparison to non-Hispanic white participants, Filipinos had a 93% greater risk of albuminuria, other Asian Americans had a 35% greater risk, and African Americans had a 22% greater risk. Furthermore, the authors speculated that crude estimates could have underestimated true effect sizes because of negative confounding. They further speculated that racial/ethnic differences in the risk of albuminuria may be attributable to unmeasured, underlying genetic or biologic differences, long-term exposures to poor risk factor control, or other environmental factors not measured in the study. Similar results were found in the study by Mau et al, in which the percentage of Filipinos with abnormal microalbuminuria (>30 mg/g) was significantly greater than the percentage among other racial/ethnic groups, and Filipinos older than 65 with diabetes were more likely than Native Hawaiians, Chinese, Japanese, and non-Hispanic whites to have CKD (7). 

It is important to continue to explore and examine large cohorts or longitudinal studies of Native Hawaiian and Asian American subgroup populations, especially those who are at risk for CKD, to accurately identify early-stage CKD and slow the progression of the disease process. These groups may be more vulnerable to CKD than non-Hispanic whites because of other comorbid diseases and sociodemographic factors associated with CKD. Evaluation and definition of estimated glomerular filtration rate and ACR and information on staging of CKD are particularly needed in the early stages of CKD among Native Hawaiians and Asian ethnicities. At the same time, refining data collection and measurements to improve validity and reliability of CKD outcome measures as well as defining and operationalizing baseline characteristics such as race/ethnicity are needed.

Our study has several limitations. First, because the study had a cross-sectional design, our findings did not confirm directionality nor causality of associations. Second, because data on sociodemographic characteristics and comorbidity were self-reported by participants, our results may have been affected by biases. Participants who self-reported race/ethnicity and self-selected to participate in this study may not be representative of the general population. Third, we also did not collect data on medications, nor management of hypertension, diabetes, obesity, cardiovascular disease, or CKD; these data could have provided additional objective information. ACR was based on a single assessment of a urine specimen; the data resulting from these tests may have been misclassified or influenced by non–kidney-related factors (eg, low muscle mass). We also did not collect data on genetic or environmental factors or dietary habits and lifestyle.

Our population health study has numerous strengths. It was a cohort study conducted by the NKFH organization during a 10-year period among a large sample of Native Hawaiians and Asian Americans. Data on these racial/ethnic groups were disaggregated, and we had detailed information on sociodemographic characteristics, anthropomorphic measurements, and comorbidities.

Using population health data to address gaps in research on racial/ethnic minority health and health disparities can lead to policy development to improve outcomes among racial/ethnic minority populations. It is important to continue to examine disaggregated data of racial/ethnic minority populations to target culturally appropriate screening, detection, referral, and intervention efforts. The findings in our study also support the need to address the disparity of knowledge and understanding of people in racial/ethnic minority populations during the early stages of CKD. Ultimately, we hope that identifying racial/ethnic-specific risk factors for CKD and targeting interventions to educate and delay disease progression among Native Hawaiians and Asian Americans (particularly Filipinos) in the United States will improve health outcomes among these groups.
